# miRNA Pathway Alteration in Response to Non-Coding RNA Delivery in Viral Vector-Based Gene Therapy

**DOI:** 10.3390/ijms232314954

**Published:** 2022-11-29

**Authors:** Darya A. Savenkova, Aelita-Luiza A. Makarova, Igor K. Shalik, Dmitry V. Yudkin

**Affiliations:** State Research Center of Virology and Biotechnology “Vector,” Federal Service for Surveillance on Consumer Rights Protection and Human Well-being (FBRI SRC VB “Vector”, Rospotrebnadzor), Koltsovo 630559, Russia

**Keywords:** miRNA pathways, adeno-associated virus, adenovirus, lentivirus, vector, gene therapy, non-coding RNA

## Abstract

Gene therapy is widely used to treat incurable disorders and has become a routine procedure in clinical practice. Since viruses can exhibit specific tropisms, effectively penetrate the cell, and are easy to use, most gene therapy approaches are based on viral delivery of genetic material. However, viral vectors have some disadvantages, such as immune response and cytotoxicity induced by a disturbance of cell metabolism, including miRNA pathways that are an important part of transcription regulation. Therefore, any viral-based gene therapy approach involves the evaluation of side effects and safety. It is possible for such effects to be caused either by the viral vectors themselves or by the delivered genetic material. Many gene therapy techniques use non-coding RNA delivery as an effective agent for gene expression regulation, with the risk of cellular miRNA pathways being affected due to the nature of the non-coding RNAs. This review describes the effect of viral vector entry and non-coding RNA delivery by these vectors on miRNA signaling pathways.

## 1. Introduction

Gene therapy is a complex approach to treating diseases by use of genetic modification. Various ways to treat genetic defects and the term “gene therapy” were proposed by Theodore Friedmann and Richard Roblin in 1972 in “Science” [1]. They suggested an exciting future for gene therapy and described some attempts at genetic modification of mammalian cells, including viral modification by an inactivated herpes simplex virus. Today, 50 years after the first mention of gene therapy, more than fifteen therapies registered in Russia, China, Europe, and the United States can be classified as gene therapies. The development of such approaches allowed one to treat previously untreated disorders, such as spinal muscular atrophy type I, beta-thalassemia, age-related macular degeneration, various types of cancer, and others [2,3,4]. The fact that viral vectors have become an essential part of gene therapy due to their natural abilities to infect mammalian cells and deliver genetic material effectively is apparent in the approved technologies and to individuals undergoing clinical trials [5]. To date, according to clinicaltrials.gov [3], there are 285 therapeutic trials based on vectors of AAV (adeno-associated virus), 234 trials based on AdV (adenovirus) vectors, 61 trials based on LVV (lentiviral vector), and few trials based on other viral vectors such as vaccinia, VSV (vesicular stomatitis virus), and others. Therefore, AAVs, especially rAAV (recombinant AAV), AdV, and LVV, are becoming the basic therapeutic delivery systems because of their usability and safety.

AAV is a small non-enveloped virus from *Parvoviridae* with a 4.7 kb linear ssDNA (+) or (−) genome, requiring helper viruses such as AdV, herpesvirus, and some others for replication. The absence of helper viruses can lead to AAV integration into the human genome via a specific integration site [6]. Four genes, *rep*, *cap*, *aap*, and *maap*, are flanked by the viral ITR (inverted terminal repeat). When AAV is used as a vector, these genes are replaced by the gene of interest by the viral genome, with a size of no more than 4.7 kb [7]. Tissue specificity of more than ten serotypes should be taken into account when using AAV as a vector for gene therapy. Different serotypes use a variety of primary entry receptors, causing the viruses to exhibit specific tropisms [8]. For example, AAV8 has been shown to efficiently transduce in the livers of rodents and non-human primates [9], while AAV1 and AAV9 can effectively deliver genes to skeletal and cardiac muscles in various animal models [10,11,12,13,14,15]. AAV is a natural human symbiont, and a weak immune response makes it a suitable vector for gene therapy [7,16,17]. Although expensive, three rAAV-based gene therapies are currently available, such as Luxturna^®^, Glybera^®^, and Zolgensma^®^ for treating Leber congenital amaurosis, lipoprotein lipase deficiency, and spinal muscular atrophy type I, respectively [18]. Despite the widespread use of AAVs in clinical practice, some problems can arise in response to AAV-based therapy, such as an immune response and immune-mediated toxicities. These adverse effects are due to both the high homology with the wild-type virus and the vector dose and design [19].

AdV is a non-enveloped virus with a linear dsDNA genome belonging to *Adenoviridae*, with its genome size ranging from 26 to 46 kbp and containing protein-coding genes [20,21]. It can cause gastroenteritis, conjunctivitis, cystitis, and respiratory diseases. AdVs can infect both replicating and quiescent cells and do not naturally integrate their DNA into the host cell genome [22]. Nowadays, vectors based on adenoviruses have found wide applications both in gene therapy and vaccine development because of their high levels of gene expression, transduction efficiency, and ability to deliver transgenes of up to 8 kbp in size [22,23]. In addition, there are HC-AdV (high-capacity vectors based on adenoviruses) with a capacity of up to 37 kb [24]. AdVs were studied from the perspectives of developing vaccines against various infectious diseases, including HIV, influenza virus, ebolavirus, SARS-CoV-2, and others [20,25,26,27], as well as gene therapy for cancer [20,22,28]. AdV-based gene therapy clinical trials account for almost half of the viral-based vector trials worldwide [3,20]. Therefore, studying the patient response to AdV-based vectors is becoming increasingly relevant.

Lentivirus is an enveloped virus belonging to the family *Retroviridae*. Most lentiviral vectors are based on the human immunodeficiency virus type 1. The HIV-1 genome is a single-stranded (−) RNA molecule of 9.2–9.6 kB in length [29]. An HIV-1-derived vector represents several plasmid-expression systems, containing a transfer vector and plasmids encoding HIV proteins. The segregation of viral components is required to deliver LVVs into cells. Lentiviral vectors are widely applied in gene therapy because of their various advantages, such as a packaging capacity of up to 10 kb [30], low cytotoxicity, and lack of viral replication after transduction. LVVs possess a genome-integration ability and ensure stable and long-term transgene expression. However, LVVs have some potential problems, such as insertion mutagenesis or transactivation of nearby genes and generation and propagation of replication-competent viral particles [31]. First- and second-generation lentiviral vector systems pose a high risk of oncogenesis due to their insertion ability [32,33,34,35,36], but third- and fourth-generation vectors have a modified LTR (long terminal repeat) and are considered to be safe [37].

Despite safety reports appearing for commercial reasons, gene therapy, similar to many other medicines or therapies, has side effects, especially when viral vectors are used [38,39,40]. Side effects can be caused by different aspects of cell metabolism, including effects on miRNA pathways that are an important part of cell metabolism regulation. Using viral vectors can cause two types of side effects: a response to the virus’s entry or a reaction to the delivered genetic material. Cellular stresses, such as viral infections, are known to affect the expression levels of various miRNAs [41,42,43]. Gene therapy, based on the delivery of non-coding RNAs via viral vectors, has proven to be effective. For example, there have been several successful cases of treating HCV (Hepatitis C Virus) infection, Huntington’s disease, and HIV-1 infection with the delivery of non-coding RNAs by AdV, AAV, and LVV, respectively [44,45,46,47]. The delivery of therapeutic non-coding miRNAs using AAV, LVV, and AdV has several advantages, such as the effective transduction of target cells and specific tropism. However, several studies have reported the endogenous miRNA expression profiles to be altered after transfection with viral vectors [48,49]. In addition, the cellular immune response to a virus involves the activation of miRNA pathways [50]. Transgene penetration, especially of non-coding RNAs, leads to gene expression profile alterations that are also interconnected with miRNA regulation because these molecules are essential for gene expression regulation [51]. This review describes the known cases of dysregulation and alteration of miRNA pathways in response to gene therapy involving the most common viral vectors based on adeno-associated virus, adenovirus, and lentivirus.

## 2. miRNA Pathway Dysregulation in Response to the Entry of Genotherapeutic Viruses

### 2.1. Adeno-Associated Virus

**Figure 1:**
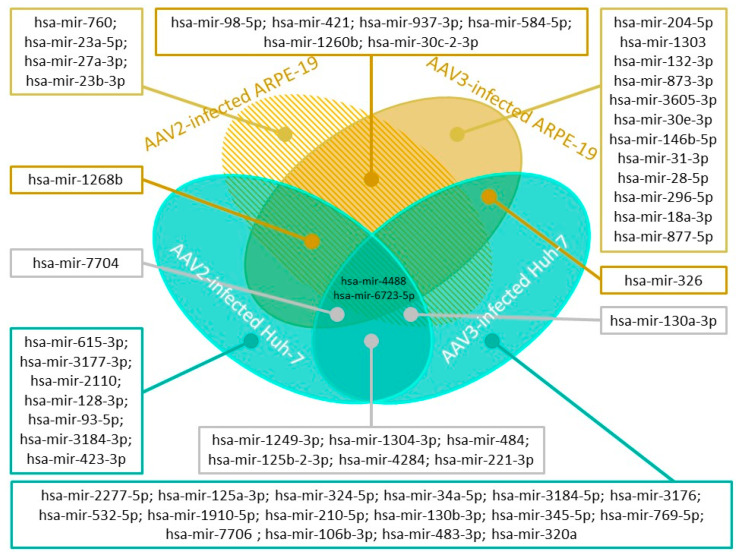
Example of the complexity of endogenous miRNA pathway alterations in AAV infections depending on the cell line and AAV serotype. This figure shows cell line-specific miRNAs, AAV serotype-specific miRNAs, and general miRNAs that are presented independently on the cell line and AAV serotype.

AAV is considered to be a safe vector for gene therapy, although any viral infection leads to cellular responses, including miRNA pathway disruption. Most DNA viruses, including AAV, are well-known expressers of miRNAs [52,53]. AAV infection was shown to induce miRNA-mediated changes in the host cell cycle. A small RNA-seq analysis of AAV2-infected HeLa cells revealed no changes in the expression profiles of endogenous miRNA but demonstrated the existence of viral miRNAs, such as sR-108, sR-271, and sR-1862, which regulate host cell metabolism and helper virus activity, and predicted sR-1, sR125, and sR-141. The alignment with other AAV serotype genomes revealed high conservation for sR-108 and sR-1862, except for AAV5 [54]. Later, a detailed analysis revealed AAV2’s ability to induce changes in the expression of fourteen miRNAs in the ARPE-19 cells and eighteen miRNAs in Huh-7 cells. Additionally, AAV3 was shown to induce changes in the expression of twenty-three miRNAs in ARPE-19 and twenty-seven miRNAs in Huh-7. Some of these miRNAs are specific, while others are common to all cell lines and AAV serotypes, making the alteration of miRNA pathways complex and dependent on various parameters (Figure 1) [55].

**Figure 2:**
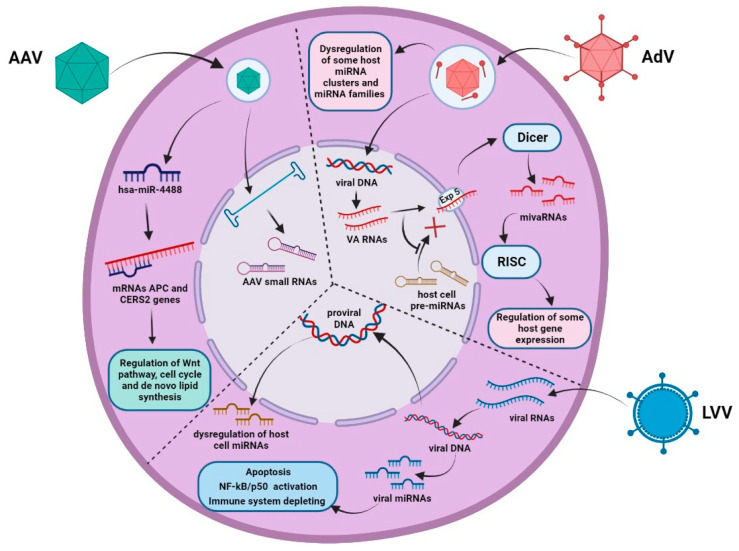
Scheme of miRNA pathway dysregulation by AdV, AAV, and LV infections. Description is within the text. (Created in BioRender.com).

Among the gene regulators, one important miRNA is hsa-miR-4488, with its downregulation detected in all AAV-infected samples of Huh-7 and ARPE-19 cell lines (Figure 2). Specifically, hsa-miR-4488 is known to target the *APC* and *CERS2* genes. *APC* is a tumor suppressor gene, and the APC protein binds to the beta-catenin complex, leading to negative regulation of the Wnt signaling pathway. In addition, this protein is involved in regulating cell adhesion, chromosome segregation, and the cell cycle. CERS2 is an ER synthase enzyme involved in de novo lipid synthesis [55,56]. Another important gene regulator that is downregulated five-fold in response to an AAV infection is mir-3687. TargetScan predicted this miRNA to be a possible target of *MTA2* (metastasis associated with family 1, member 2), which is responsible for the nucleosome reconstruction of the deacetylase complex in the nucleus.

The study of miRNA pathway dysregulation in response to an AAV infection revealed two novel miRNAs expressed in Huh-7 and ARPE-19 samples only in the presence of AAV. The first miRNA was found to be commonly expressed in AAV3-infected Huh-7 and ARPE-19 cells, and the second was detected only in Huh-7 cells infected with AAV2 and AAV3. The first miRNA was localized in the region of the *SMAD3* gene transcript, and the second miRNA was derived from *UCKL1* antisense RNA [55].

The fact that AAV, despite its non-pathogenicity, can dysregulate a range of miRNAs that may be responsible for important cellular processes must be taken into account by researchers developing gene therapy approaches with AAV vectors.

### 2.2. Adenovirus

AdV infection affects multiple cellular processes, including endogenous miRNA pathways. A striking example of such an impact is the alteration of host miRNAs by AdV VA RNAs (viral-associated RNAs) (Figure 2). There are two types of VA RNAs: VA RNAI and VA RNAII. VA RNA synthesis begins at the early phase of infection, with VA RNAI and VA RNAII reaching 10^8^ and 10^7^ molecules per cell by the late phase, respectively [57]. The main function of VA RNAI is to inhibit the PRKRA (dsRNA-activated protein kinase) to avoid the antiviral effects of IFN (interferon) during the viral infection [58]. In addition, VA RNAI interacts with other dsRNA-detecting proteins, such as RAI1 and 2′-5′ oligoadenylate synthase-1 (OAS1). RAI1 is a cytosolic receptor that recognizes pathogen-associated molecules, such as dsRNA or 5′-triphosphorylated RNA, and activates the downstream signaling pathway to induce IFN type I activation [59]. OAS1 polymerizes ATP into 2′-5′ oligoadenylate, leading to ribonuclease activation and mRNA degradation [60]. In addition, VA RNAs can interfere with endogenous miRNA pathways, resulting in miRNA activity disruption [61]. After the transcription by cellular RNA polymerase III, VA RNAI is exported to the cytoplasm by Exportin 5, which is also responsible for cellular pre-miRNA transfer from the nucleus. Competition between cellular miRNA precursors and VA RNAs for the export from the nucleus results in rapid saturation of Exportin 5 and blocks the pre-miRNA export to the cytoplasm [62]. Moreover, with Exportin 5 providing the export of Dicer mRNA, the saturation of this transport pathway decreases the amount of Dicer protein in the cytoplasm [63]. In addition, VA RNAI interferes with Dicer and prevents its binding to the host cell pre-miRNAs [62]. After being transported across the nuclear membrane, VA RNAs are processed by Dicer and generate smaller RNA molecules called mivaRNAs (VA RNA-derived small RNAs). Then, mivaRNAs are loaded into RISC (RNA-induced silencing complex) and can act as cellular miRNAs, repressing the expression of some genes by binding with the 3′UTR of host mRNAs. In particular, these mivaRNAs are involved in regulating the genes responsible for cell growth, gene expression, and DNA repair [64,65]. MivaRNAII, which is VA RNAII-derived, is associated with polyribosomes, indicating that they are involved in regulating the host cell’s mRNA translation [66]. Therefore, VA RNAs use the pathways of cellular miRNA maturation and eventually disturb it, resulting in miRNA profile alteration.

AdV VAI is a precursor of mivaRI-138, which is responsible for repressing the *TIA-1* gene encoding RNA binding proteins, which is important for splicing regulation and translation repression [67].

Different changes in the expression profiles of several miRNA clusters were observed depending on the AdV serotype and the stage of infection. In response to the AdV2 infection, two miRNA clusters exhibited the most significant alteration in the expression levels. Thus, miRNAs from miR-17/92 and miR-23a/27a clusters were highly expressed upon infection but at various periods: early and late phases of infection, respectively. The expression of miR-17-5p, miR-18a-5p, and miR-20a-5p was observed to decrease more than 1.5-fold, whereas that of hsa-miR-19a-3p increased 1.7-fold 12 h after infection. Also, the expression profiles of miRNAs from let-7, mir-10, mir-17, mir-30, and mir-320 families were found to be altered. Remarkably, during the early phase of viral infection, miRNAs known as tumor suppressors were upregulated, while oncogenic miRNAs were inhibited. However, in the late stage of AdV infection, an upregulation of oncogenic miRNAs and a tumor suppressor miRNA downregulation on the late AdV infection phase were observed [68]. For AdV3, changes in many different miRNAs have been demonstrated to occur during infection in Hep2 (Human laryngeal epithelial) cells: 44 miRNAs (hsa-miR-1974, hsa-miR-1975, hsa-miR-7, and others) had higher, and 36 miRNAs (hsa-miR-27b, hsa-miR-125b, hsa-miR-27a, and others) had lower expression levels compared to the non-infected controls [69]. Recent studies have shown that individuals who were previously infected by AdV36 had higher expression levels of the pro-adipogenic hsa-miR-17. In addition, a decrease in anti-adipogenic hsa-miR-155 was observed. AdV36 infection was found to positively affect adipose cell differentiation, with proliferation and dysregulation of hsa-miR-17 and hsa-miR-155 caused the increased adipogenic status in AdV36-serotype-positive individuals [70].

Endogenous miRNAs can also influence the AdV life cycle through various mechanisms. For instance, hsa-miR-27a/b is known to suppress the expression of SNAP25 (synaptosomal-associated protein, 25 kDa) and TXN2 (thioredoxin-2). The inhibition of SNAP25 and TXN2 prevents AdV’s entry into cells and its replication, respectively. Therefore, hsa-miR-27a/b overexpression leads to efficient inhibition of adenovirus infection [71]. Another example of host cell miRNAs affecting the development of AdV infection is hsa-miR-26b. In the case of human AdV5 infection, overexpression of this miRNA results in increased AdV-mediated cell death, viral propagation, and spread in some prostate cancer cell lines by inhibiting the AdV-induced Nuclear Factor kappa B (NF-kB) activation. This property of hsa-miR-26b makes it a possible helper for the more effective treatment of prostate cancer with oncolytic AdVs [72].

To sum up, AdV infection has a significant influence on the levels of endogenous miRNAs. It is evident that understanding the mechanisms of interaction between AdVs and infected cells is essential for both preventing AdV infections and creating effective and safe adenovirus-based vectors. Knowledge about the dysregulation of endogenous miRNA pathways and changes in their expression profiles in response to adenoviruses may reveal new strategies for improving AdV vectors.

### 2.3. Lentivirus

Lentivirus entry induces the dysregulation of multiple miRNA pathways (Figure 2). In particular, HIV-1 infection affects various miRNA signaling pathways associated with immune response [73], neuropathogenesis [74], apoptosis, cell proliferation [75], and others.

The expression profiles of miRNAs are altered during HIV-1 infection. In clinical applications, miRNAs are used as biomarkers for HIV-1 diagnostics and therapy efficacy [76,77]. The change patterns depend on various parameters. An extensive analysis of miRNA expression profiles in PBMCs (peripheral blood mononuclear cells) of HIV-1-infected patients revealed the differences in expression patterns depending on the quantity of T-cells and viral load. Sixty-two miRNAs with altered expression were detected. Additional experiments with PBMCs directly infected with HIV-1 revealed some changes in the miRNA expression profile. Immune response activation causes significant changes in miRNA expression profile, while immune response suppression during HIV-1 infection results in changes in the expression of only one miRNA. For example, hsa-miR-223 and hsa-miR-150 target the HIV-1 genome and suppress viral replication, with changes in the expression profiles of these miRNAs varying in patients with different viral loads and numbers of T-cells [73].

A possible anti-HIV protective miRNA is hsa-miR-31, which targets *STAT1*. This miRNA is involved in maintaining T-cell homeostasis, and decreased levels of this miRNA lead to increased susceptibility of cells responsible for HIV-1 infection [78].

Cells expressing other miRNAs target major HIV-1 genes, such as *Vpr*, *Env*, *Viv*, and UTRs, or activate the *Tat* inhibitory pathways. The examples of miRNAs described are hsa-miR-149, hsa-miR-378, hsa-miR-324-5p, hsa-miR-191-5p, hsa-miR-27b, hsa-miR-29b, and others [75,79,80,81,82,83,84,85,86].

HIV-1 infection is known to induce increased expression of human hsa-miR-186, -210, and -222 targeting Dicer-1, HIV-EP2 (HIV-1 Enhancer Binding Protein 2), and HRB (HIV-1 Rev-binding protein). Due to the high expression of hsa-miR-186, -210, and -222, these three genes were silenced, leading to infection latency in Sup-T1 cells. A possible mechanism of action is related to Dicer-1 being necessary for HIV-TAR miRNA processing and HIV-EP2 and HRB being equally crucial for the HIV-1 lifecycle [87].

The HIV-1 Vpr protein can regulate the cell cycle by interacting with endogenous miRNA pathways. Vpr activates the NF-kB/p50 signaling pathway, which is followed by the upregulation of hsa-miR-210-5p expression. This miRNA targets the TGIF2 transcription factor and downregulates its level, resulting in G2 arrest. G2 arrest contributes to enhanced HIV-1 replication and viral production and can cause apoptosis of HIV-infected T-cells, potentially depleting the immune system [88].

The fact that the lentivirus itself and the empty lentiviral vector can induce alterations in endogenous miRNA pathways was at least shown for anti-HCV miRNAs, such as miR-217 and miR-216a-5p, that increased their expression in response to LVV [49].

The lentivirus genome encodes several miRNAs that regulate viral and host gene expression. Two miRNAs derived from the trans-activation response element, referred to as miR-TAR-5p and miR-TAR-3p, were described as regulators of host gene expression through association with Ago protein complexes [89,90,91]. Another miRNA encoded in the HIV genome is hiv1-miR-H1, shown to be able to induce cell apoptosis through the “epigenomic pathway” by downregulating AATP gene expression followed by suppressing genes coding for Bcl-2, c-myc, Par-4, and Dicer. In addition, hiv1-miR-H1 downregulated the cellular hsa-miR-149 known to target the HIV-1 Vpr protein, thus helping the virus avoid a cellular antiviral response [92]. HIV-1 gene expression is regulated by miR-H3, which interacts with the TATA box in the 5′LTR of the viral genome and upregulates the viral genome replication and viral gene expression. miR-H3 is encoded by the *pol* gene region, and its sequence is highly conservative among different subtypes [93]. Two miRNAs encoded in the HIV-1 LTR, vmiR88, and vmiR99, are not involved in RNA interference and play essential roles in exocytosis to transfer miRNAs into uninfected cells and induce a chronic immune response through TNFα activation [94]. In addition, miR-N367 was detected in AIDS patients who are long-term non-progressors (LTNP). This miRNA is derived from HIV-1 *nef* and leads to low viremia in LTNP [95].

In summary, lentivirus and LVV transduction can cause side effects on miRNA profiles, influence the cell cycle through miRNA expression changes, and express their own miRNAs that could help the virus in its life cycle.

## 3. Examples of miRNA Pathway Dysregulation in Response to Non-Coding RNA Delivery

There is a wide range of non-coding RNAs, some of which can be used as therapeutic agents, with their nature allowing them to interact with endogenous miRNA pathways.

One of the therapeutic agents currently in use is shRNA (short hairpin RNA), an alternative regulatory RNA interference effector that can be delivered by various viral vectors. shRNA is a short hairpin RNA used for gene silencing by RNA interference through binding with the target mRNA [96]. Using such regulators was demonstrated to cause significant changes in miRNA expression profiles. The main problem is competition with endogenous miRNAs for processing proteins. shRNAs are known to mimic miRNA biogenesis intermediates at various stages and must be processed by the miRNA maturation system to be loaded into RISC to be functional. Relatively large amounts of transfected shRNAs can compete for Ago proteins and, thus, disrupt endogenous miRNA function, potentially leading to toxicity [97]. The toxic effect depends on shRNA overexpression caused by the PolIII promoter and can be eliminated using alternative promoters [49,98]. The toxic effect on the liver was detected when using rAAV-shRNA. Hepatotoxicity occurs when exogenous shRNAs exceed 12% of the total liver miRNAs by specifically reducing the miR-122-5p 22 bp isoform without significantly affecting other miRNAs, resulting in a functional depression of target miR-122 mRNAs [99]. The same decrease in miR-451 was detected in the heart in response to shRNA delivery. The toxic effect depends on the AAV serotype used because of its tissue specificity [100]. The same effects were observed in other studies of gene-specific shRNAs [101,102,103].

shRNAs were demonstrated to be effective in inhibiting HIV-1 replication. However, there are two significant problems. The first one is the negative impact of shRNAs on the maturation pathways of endogenous miRNAs, described above. This problem can be solved using shRNAs containing pri-miRNA processing signals expressed under PolIII or PolII promoters. The second problem is the rapid emergence of escape mutations of HIV-1, which can be resolved using the shRNA cassette against host factors and viral genes [46].

Pseudotyped HIV-1 lentivirus expressing seven shRNAs against *CCR5*, *Gag*, *Env*, *Tat*, *Pol*, and *Vif* in a polycistronic miRNA backbone was reported to maintain effective suppression of HIV-1 replication in Hu-PBL mice. Moreover, this technique ensures that each shRNA is sufficiently expressed to suppress HIV-1 infection and avoid the dysregulation of endogenous miRNA function [46].

Another therapeutic agent with activity similar to shRNAs is pri-amiRNA (primary artificial miRNA), which can be delivered by AAV. In Huntington’s disease therapy development, pri-amiRNAs delivered by AAV did not affect the profile of endogenous miRNAs, making this approach preferable to prevent side effects [47].

The helper-dependent AdV (HD AdV) expressing pri-miRNAs that mimic HBV was reported to sufficiently suppress HBV replication in mice without off-target effects and toxicity [104].

A novel strategy for the knockout of highly specific miRNA genes, which is comparable in efficiency to the shRNA approach, is the delivery with LVV of dCas9-KRAB-shRNA and sgRNA. This strategy allows one to silence specific miRNA genes with no effect on endogenous miRNA pathways, even in the case of high homology to the miRNA sequence, making dCas9-KRAB an alternative for shRNA [105].

Another example of the therapeutic use of non-coding RNAs is miRNA regulation to reduce the toxicity of viral vectors. In oncolytic AdV, the miRNA binding site is located in the 3′UTR of the E1A viral sequence. Using a specific binding site depends on target cells for AdV to provide degradation of AdV in non-target cells by miRNA [106,107]. For example, miR-216a and miR-148a target binding sites allow replication of oncolytic AdV in PDAC (pancreatic ductal adenocarcinoma) cells only and do not influence any cellular miRNA pathways [108]. The same results were obtained for the miR-122 binding site and some other miRNA binding sites [109,110,111]. Thus, the endogenous miR-122-regulated oncolytic AdV does not influence the cell miRNA biogenesis pathway. However, in theory, inhibition of AdV genome materials by the RNA interference mechanism could occur, potentially causing the dysregulation of endogenous miRNAs. Cawood et al. emphasized that using other miRNAs to regulate AdV may cause insufficient viral inhibition in normal cells, possibly leading to a competition between endogenous and exogenous AdV mRNAs for binding with target miRNAs [111]. A recent study has suggested negligible dysregulation of endogenous miRNAs and limitation of the potential inhibition of host cell miRNA biogenesis pathways through AdV replication and VA-RNA expression [112].

The regulation by endogenous miRNAs was found not only for AdV vectors. This strategy is widely used for AAV-based transgene delivery [113,114,115,116]. Endogenous miR-206 was documented to be expressed in skeletal muscle cells but not in the heart. For selective silencing of miR-206-regulated heart-specific AAV9 in skeletal muscle cells, the miR-206 binding site was integrated into the 3′UTR of AAV9. However, the suppression of AAV9 was observed both in skeletal muscle and heart cells because miR-1 expressed in the heart cells can interact with the miR-206 binding site. Inserting a mismatch mutation into the miR-206 binding site can provide specific binding of miR-206 to this target site but not to miR-1. The mutated miR-206 target site supports the selective activity of AAV9 in heart cells and silencing in liver and skeletal muscle cells [117].

Nevertheless, an exogenous transgene regulated by the endogenous guide miRNA strand can act similarly to a miRNA-sponge and compete with the cellular target gene of miRNA. The abundance of the transgene with the binding site for the guide miRNA strand can lead to the attenuation of endogenous miRNA activity [118,119,120]. The endogenous miRNA passenger strand can be used to eliminate these effects [120]

One more approach in non-coding RNA usage for therapy is to suppress several endogenous miRNAs for effective treatment, which can be achieved using a viral vector. For example, liver-specific miRNA-122a facilitates the replication of HCV in infected cells. Effective HCV replication suppression was reached by using AdV expressing TuD (ToughDecoy)-RNA against miR-122a (TuD-122a) [44,45]. Similar results were obtained by suppressing miR-21 with an AdV-based vector (Ad-TuD-21) coding the sponging TuD-RNA against miR-21 [45,121]. The same anti-miR-21-TuD delivered by rAAV8 was used to treat IL-13-mediated schistosomiasis liver fibrosis, causing the increased miR-21 levels. The data obtained confirmed a significant decrease in hepatic miR-21, but not in miR-122, in mice treated with the rAAV8-anti-miR-21-TuD vector [122].

However, sometimes the development and progression of pathological conditions are associated with the dysregulation of many endogenous miRNAs. For example, hepatocellular carcinoma (HCC) cells demonstrate significant changes in the expression profiles of several oncogenic miRNAs (OncomiRs), such as miR21, miR221/222, miR224, miR17-5p/20a, miR10b, miR106b, miR151-5p, miR155, miR181a/181b, and miR184. The AdV-based vector carrying an artificial interfering long non-coding RNA (lncRNAi) against 12 oncomiRNAs has demonstrated decreased proliferation and invasion levels and caused apoptosis of tumor cells due to the inhibition of endogenous target oncomiRNAs in HCC cells [123].

Given the above, the abundance of non-coding RNA gene therapy approaches and the diversity of types of alterations in endogenous miRNA pathways is evident. This diversity cannot be addressed in a single paper. However, this brief overview allows a general concept to be shaped.

## 4. Conclusions

Viral vectors are widely used for non-coding RNA delivery to cells as therapeutic gene approaches. We have described the usage of AAV, AdV, and LVV as delivering agents. Several studies have shown that each virus described can induce cellular miRNA pathway alterations. For example, AAV infection leads to the downregulation of hsa-miR-4488, resulting in changes in the expression of important genes. Adenovirus has non-coding RNAs that saturate miRNA processing pathways, influencing cell metabolism. Lentiviral Vpr protein induces dysregulation of the miRNA responsible for regulating TGIF2 transcription factor expression level. These are only some changes induced by the viral infection. The same effects can be seen for viral vectors containing parts of the viral genome and viral protein in capsids.

However, the most expressed effects on endogenous miRNA pathways have been described for non-coding RNAs delivered by viral vectors analyzed in this review. These effects strongly depend on the type and sequence of the delivered material and cannot be extrapolated to every non-coding RNA-based therapeutic approach.

All gene therapy techniques described have significant advantages, but numerous studies have demonstrated their side effects on cellular metabolism, including the miRNA pathways. Therefore, developing gene therapies, especially those using viral vectors, requires a detailed study of their effects on cellular processes, including gene expression regulation by miRNA.
